# Design, Practical Synthesis, and Biological Evaluation of Novel 6-(Pyrazolylmethyl)-4-quinoline-3-carboxylic Acid Derivatives as HIV-1 Integrase Inhibitors

**DOI:** 10.3390/molecules170910652

**Published:** 2012-09-06

**Authors:** Liming Hu, Song Yan, Zaigang Luo, Xiao Han, Yujie Wang, Zhanyang Wang, Chengchu Zeng

**Affiliations:** 1 College of Life Science and Bioengineering, Beijing University of Technology, Beijing 100124, China; 2 College of Chemical Engineering, Anhui University of Science Technology, Huainan 232001, China

**Keywords:** HIV-1, integrase inhibitors, quinolone-3-carboxylic acid derivatives

## Abstract

A series of novel 6-(pyrazolylmethyl)-4-oxo-4*H*-quinoline-3-carboxylic acid derivatives bearing different substituents on the N-position of quinoline ring were designed and synthesized as potential HIV-1 integrase (IN) inhibitors, based on the structurally related GS-9137 scaffold. The structures of all new compounds were confirmed by ^1^H-NMR, ^13^C-NMR and ESI (or HRMS) spectra. Detailed synthetic protocols and the anti-IN activity studies are also presented.

## 1. Introduction

**Figure 1:**
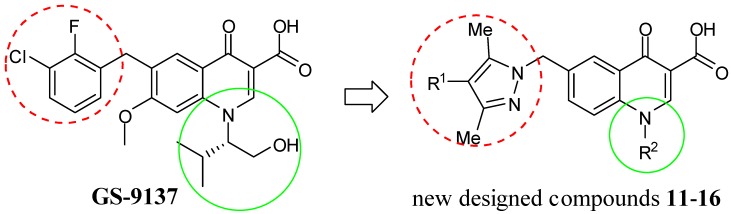
Structure of GS-9137 and new 6-(pyrazolylmethyl)-4-oxo-4*H*-quinoline-3-carboxylic acid derivatives as potential HIV in inhibitors.

Human immunodeficiency virus type 1 (HIV**-**1) encodes three enzymes reverse transcriptase, protease, and integrase which have been identified as key targets to arrest the HIV life cycle. To date, most of the currently used oral drugs for the treatment of HIV infection inhibit the first two of these enzymes. Unfortunately, the infection has been difficult to cure because of drug resistance and immune response [1]. The viral enzyme integrase (IN), which is essential for interrupting the viral replication cycle and has no counterpart in mammalian cells, is a crucial target for the development of new HIV-1 inhibitors with high selectivity and low toxicity [2,3]. As a kind of HIV-1 inhibitors, quinolone derivatives have great attractive to researcher because of their small extremely versatile molecules, easily synthesized at low cost on a large scale and endowed with well-known biochemical properties [4,5]. Researchers at Japan Tobacco reported the quinoline-3-carboxylic acid GS-9137 (elvitegravir, Figure 1), which has finished phase III trials [6].

**Figure 2:**
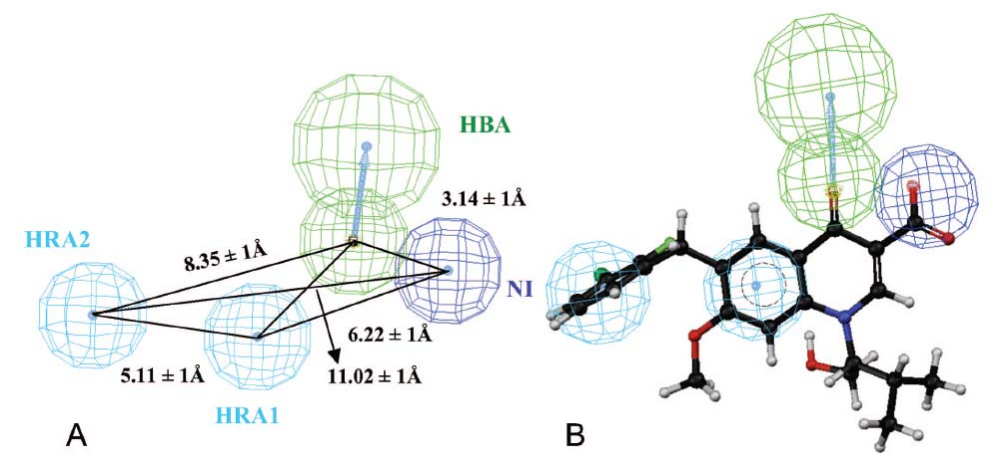
(**A**) 3D arrangement of the four pharmacophoric features in the quinolone 3-carboxylic acid pharmacophore. (**B**) The clinically studied HIV-1 integrase inhibitor (GS-9137) is mapped onto the quinolone 3-carboxylic acid pharmacophore. The pharmacophore features depicted as H-bond acceptor (HBA) in green, negatively ionisable (NI) feature in blue and hydrophobic features (HRA1-2) in cyan. Interfeature distances are given in angstroms.

Gilead submitted a marketing application to the U.S. FDA for elvitegravir on Jun 27, 2012. Quinoline-3-carboxylic acid can be used as an alternative scaffold to diketo acids (DKA) in order to obtain a novel class of IN inhibitors through modification of antibiotic quinolones [7]. High interest were generated by this class of inhibitors to study their structure-activity relationships (SAR) [8,9,10,11]. The quinoline-3-carboxylic acid pharmacophore was developed utilizing a training set of compounds including GS-9137 [8] (Figure 2).

The pharmacophore models of these compounds have four distinct characteristics: a negatively ionizable (NI) feature, an H-bond acceptor (HBA) feature and two hydrophobic aromatic (HRA) features, mapping of the hypothesis onto the GS-9137 shows an excellent agreement between key chemical features of the compound and the pharmacophoric features of the hypotheses [8]. When the methylene at the C-6 position was replaced by ethylene, carbonyl, amide, inverse amide, and amino group, the resulting compounds proved to be completely inactive (IC_50_ > 100 µM) or a loss of activity in the strand transfer (ST) assay occurred with hydroxyalkyl chains of variable length at the N-1 position [9]. Even removal of spacer between the two aromatic rings, characterized by a phenyl or a pyridyl group directly linked to C-6, proved to be completely inactive in inhibiting the enzyme integrase, thus displaying the importance of a methylene spacer [9].

On the basis of the above information as well as on our interest in the development of new potential anti-HIV agents [12], we decided to explore the effect of chemical structure modification on the quinoline-3-carboxylic acid scaffold by decorating it at the N-1 and C-6 positions, with the aim of gaining further SAR data for anti-IN activity. We prepared the final compounds **11–16** (Figure 1) based on the following considerations: (1) A large number of compounds with important biological and pharmaceutical activities contain pyrazoles as a key substructure [13,14] and its planar structure may facilitate the π stacking interaction with target enzymes similar to a phenyl ring, so it is very interesting to design novel biologically active compounds by bringing the pyrazole moieties into the quinolone-3-carboxylic acid molecular framework with a view to study their additive effect on anti-IN properties. We considered that the phenyl ring at C-6 could thus be replaced by a bioisosteric pyrazole ring; meanwhile, halogen atoms were introduced at C-4′ on the pyrazole ring with two hydrophobic methyl groups also at the C-3′ and C-5′ positions; (2) Variable substituent groups were introduced at N-1 to study the effects of alkyl and aromatic spacer groups, respectively [7]; (3) The methoxy group at C-7 was ignored, although this substituent group affects the pharmacokinetic and *in vivo* activity features, it does not influence *in vitro* binding ability, which can be seen by comparing the strand transfer inhibition IC_50_ values of GS-9137 and its 7-demethoxy derivative (7.2 nM and 8.2 nM, respectively). In this paper, a series of 6-(pyrazolylmethyl)-4-oxo-4*H*-quinoline-3-carboxylic acid derivatives bearing different substituents on the N-1 position were synthesized and characterized and their anti-IN activities were also screened.

## 2. Results and Discussion

### 2.1. Synthesis of 6-(Pyrazolylmethyl)-4-oxo-4H-quinoline-3-carboxylic Acid Derivatives

**Scheme 1:**
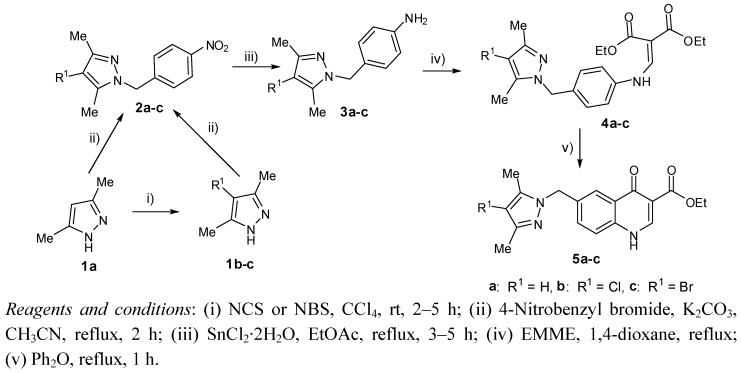
Synthesis of ethyl 6-(pyrazolylmethyl)-4-oxo-4*H*-quinoline-3-carboxylates **5**.

**Scheme 2:**
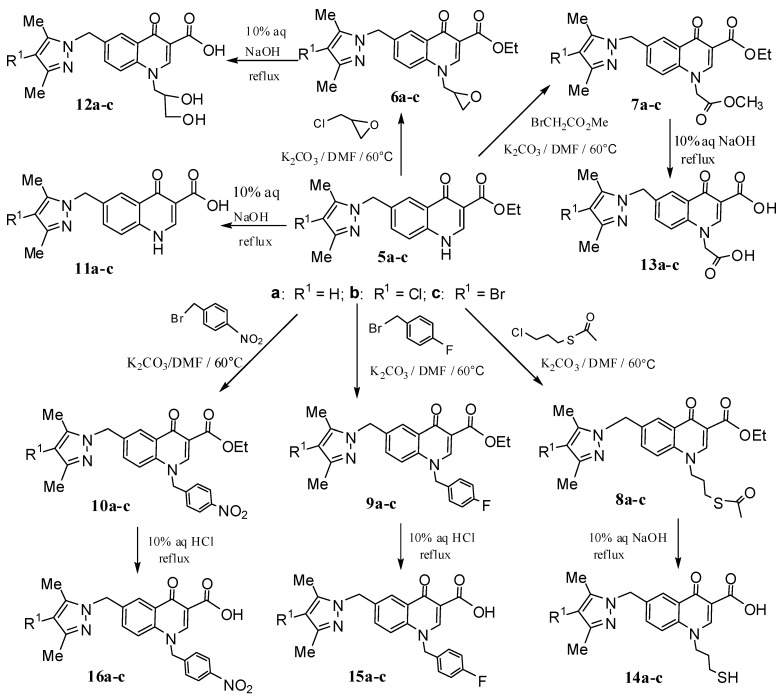
Synthesis of 6-(pyrazolylmethyl)-4-oxo-4*H*-quinoline-3-carboxylic acid derivatives **11–16**.

The syntheses of the designed 6-(pyrazolylmethyl)-4-oxo-4*H*-quinoline-3-carboxylic acid derivatives **11a–c–16a–c** are carried out as shown in Scheme 1 and Scheme 2. Compounds **1b–c**, **2a–c**, **3a–c**, **4a–c** and **5a–c** were prepared according to the corresponding literature procedures [15,16,17,18,19]. Compounds **5a–c** were then alkylated using varied alkyl halides or benzyl bromides to provide derivatives **6a–c–10a–c** in moderate yields. After hydrolysis of **5–10** in 10% aq NaOH or HCl, the corresponding 4-oxo-4*H*-quinoline-3-carboxylic acid derivatives **11a–c–16a–c** were obtained in yields ranging from 42% to 81%.

The structures of new quinoline-3-carboxylic acid derivatives **11–16** were confirmed by ^1^H-NMR, ^13^C-NMR, and ESI-MS (or HRMS) spectra. In the ^1^H-NMR spectra, one sharp proton signal for the COOH group was observed in the *δ* 14.68–15.29 range and the chemical shifts of the methylene group protons at C-6 were around 5.50 ppm, two sharp proton signals of the methyl groups at C-3' and C-5' appeared at low field (*δ* 2.08–2.27). E.g., the proton signals of the COOH group, methylene group at C-6 and the two methyl group at C-5′ and C-3′ of compound **12c** were located at 15.15 ppm, 5.51 ppm, 2.27 ppm and 2.19 ppm, respectively. The characterized protons signals at C-2, C-5, C-7 and C-8 of the 4-oxo-4*H*-quinoline ring were also clearly observed. In the ^13^C-NMR spectra of these compounds one C=O peak at about 178 ppm and one around 166 ppm corresponded to the signals of the 3-carboxylic acid and carbonyl groups in the quinoline rings. The corresponding signals of the Cl and Br isotopes in these compounds were also observed in the mass spectra.

### 2.2. HIV Inhibitory Activity

A HIV-1 integrase strand transfer (ST) activity assay was carried out to test the inhibition effectiveness of our compounds as described previously [20] with some minor modifications. Compounds diluted in DMSO were pre-incubated with 800 ng integrase at 37.8 °C in the reaction buffer in the absence of Mn^2+^ for 10 min. Subsequently, 1.5 pmol donor DNA and 9 pmol target DNA were added and the reaction was initiated by the addition of 10 mmol/L Mn^2+^ into the final reaction volume. The reactions were carried out at 37.8 °C for 1 h and subsequent detection procedure was applied to detect the assay signals. Integrase inhibitor, S-1360, was used as the control compound (positive control), whereas no compound but only DMSO in the reaction mixture was set as the drug-free control (negative control). The inhibition effects of 6-(pyrazolylmethyl)-4-oxo-4*H*-quinoline-3-carboxylic acids **11–16** were calculated based on the positive and negative controls (Table 1).

As shown in Table 1, the values of IC_50_ of these compounds are more than 100 μg/mL, which means they have a low inhibitory activity towards HIV integrase. The low HIV inhibitory activity of these designed 4-oxo-4*H*-quinolizine-3-carboxylic acid derivatives might be attributed to various cofactors. Structurally, 3′,5′-dimethyl-1*H*-pyrazolyl moiety maybe shows a reduced hydrophobic interaction ability with integrase comparable to the parent benzenyl moiety. In addition, an appropriate substituent at N-1 position seems also essential to enhance potential anti-IN activity.

**Table 1:** Integrase inhibitory activity data of 4-oxo-4*H*-quinolizine-3-carboxylic acid derivatives ^a^.

Sample	Initial concentration (µg/mL)	IC_50_ (µg/mL) ^b^
**S-1360**	7.8	0.55
**11a**	100	-c
**11b**	100	-c
**11c**	100	-c
**12a**	100	-c
**12b**	100	-c
**12c**	100	-c
**13a**	100	-c
**13b**	100	-c
**13c**	100	-c
**14a**	100	-c
**14b**	100	-c
**14c**	100	-c
**15a**	100	-c
**15b**	100	-c
**15c**	100	-c
**16a**	100	-c
**16b**	100	-c
**16c**	100	-c

^a^ HIV-1 IN inhibitory activities were measured according to the procedure described [20]. ^b^ Inhibition of strand transfer. -c indicates that the HIV-IN inhibitory effect was less than 50% at the initial concentration.

## 3. Experimental

### 3.1. General

All melting points (mp) were measured with a XT4A electrothermal apparatus equipped with a microscope and are uncorrected. ^1^H-NMR and ^13^C-NMR spectra were recorded on an AV 400 M Bruker spectrometer. Chemical shifts were measured in DMSO-*d_6_* with TMS as internal reference. The MS spectra (ESI) were recorded on a Bruker Esquire 6000 mass spectrometer. HRMS spectra were recorded in the positive ion mode using APEX IV FT-ICRMS of Bruker Daltonics Inc. Purities of target compounds **11a–c**, **12a–c**, **13a–c**, **14a–c**, **15a–c**, **16a–c** were determined by an Agilent 1260 HPLC system equipped with a Agilent Zorbax SB-C18 column (5 μm, 4.4 × 25mm), UV detector at 254 nm, mobile phase CH_3_OH/H_2_O (70%–100% or 80%) and flow rate of 1 mL/min. All solvents were of commercial quality and were dried and purified by standard procedures.

### 3.2. General Procedure for the Synthesis of Ethyl 1-Substitued-6-(pyrazolylmethyl)-4-oxo-4H-quinoline-3-carboxylates ***6**–**10***

A mixture of the appropriate quinolone derivative **5** (2 mmol), the appropriate alkyl halide or benzyl bromide (5.6 mmol), and solid K_2_CO_3_ (772 mg, 5.6 mmol) in dry DMF (5 mL) was heated under Ar atmosphere at 60 °C for 12 h and then poured into an ice-water mixture, extracted with DCM and purified by flash chromatography on silica gel eluting with DCM/MeOH (40:1–20:1).

*Ethyl6-((3,5-Dimethyl-1H-pyrazol-1-yl)methyl)-1-(oxiran-2-ylmethyl)-4-ox-1,4-dihydroquinoline-3-carboxylate* (**6a**). Yield: 33%; mp: 162–165 °C; ^1^H-NMR (CDCl_3_, *δ* ppm) 1.43 (t, 3H, *J* = 7.2 Hz, -CH_2_*CH_3_*), 2.16 (s, 3H, *CH_3_*-pyrazole), 2.24 (s, 3H, *CH_3_*-pyrazole), 2.59 (q, 1H, *J* = 4.4 Hz, *CH_2_*OCHCH_2_-), 2.92 (t, 1H, *J* = 4.4 Hz, *CH_2_*OCHCH_2_-), 3.33–3.36 (m, 1H, CH_2_O*CH*CH_2_-), 4.20 (dd, 1H, *J* = 5.6 and 15.6 Hz, CH_2_OCH*CH_2_*-), 4.42 (q, 2H, *J* = 7.2 Hz, -*CH_2_*CH_3_), 4.55 (dd, 1H, *J* = 2.0 and 15.6 Hz, CH_2_OCH*CH_2_*-), 5.32 (s, 2H, -*CH_2_*-pyrazole), 5.86 (s, 1H, *H*-pyrazole), 7.44 (dd, 1H, *J* = 2.4 and 8.8 Hz, H7), 7.50 (d, 1H, *J* = 8.8 Hz, H8), 8.32 (d, 1H, *J* = 2.0 Hz, H5), 8.43 (s, 1H, H2).

*Ethyl 6-((4-Chloro-3,5-dimethyl-1H-pyrazol-1-yl)methyl)-1-(oxiran-2-ylmethyl)-4-oxo-1,4-Dihydro- quinoline-3-carboxylate* (**6b**). Yield: 33%; mp: 200–202 °C; ^1^H-NMR (CDCl_3_, *δ* ppm) 1.42 (t, 3H, *J* = 6.8 Hz, -CH_2_*CH_3_*), 2.15 (s, 3H, *CH_3_*-pyrazole), 2.23 (s, 3H, *CH_3_*-pyrazole), 2.59 (q, 1H, *J* = 4.8 Hz, *CH_2_*OCHCH_2_-), 2.92 (t, 1H, *J* = 4.4 Hz, *CH_2_*OCHCH_2_-), 3.32–3.36 (m, 1H, CH_2_O*CH*CH_2_-), 4.19 (dd, 1H, *J* = 6.0 and 15.6 Hz, CH_2_OCH*CH_2_*-), 4.42 (q, 2H, *J* = 7.0 Hz, -*CH_2_*CH_3_), 4.56 (dd, 1H, *J* = 2.0 and 15.6 Hz, CH_2_OCH*CH_2_*-), 5.31 (s, 2H, -*CH_2_*-pyrazole), 7.44 (dd, 1H, *J* = 2.4 and 8.8 Hz, H7), 7.51 (d, 1H, *J* = 8.8 Hz, H8), 8.33 (d, 1H, *J* = 2.0 Hz, H5), 8.43 (s, 1H, H2).

*Ethyl 6-((4-Bromo-3,5-dimethyl-1H-pyrazol-1-yl)methyl)-1-(oxiran-2-ylmethyl)-4-oxo-1,4-dihydro-quinoline-3-carboxylate* (**6c**). Yield: 47%; mp: 199–201 °C; ^1^H-NMR (CDCl_3_, *δ* ppm) 1.42 (t, 3H, *J* = 7.2 Hz, -CH_2_*CH_3_*), 2.16 (s, 3H, *CH_3_*-pyrazole), 2.23 (s, 3H, *CH_3_*-pyrazole), 2.59 (q, 1H, *J* = 4.4 Hz, *CH_2_*OCHCH_2_-), 2.92 (t, 1H, *J* = 4.4 Hz, *CH_2_*OCHCH_2_-), 3.33–3.36 (m, 1H, CH_2_O*CH*CH_2_-), 4.18 (dd, 1H, *J* = 6.0 and 15.6 Hz, CH_2_OCH*CH_2_*-), 4.41 (q, 2H, *J* = 7.2 Hz, -*CH_2_*CH_3_), 4.56 (dd, 1H, *J* = 2.4 and 15.6 Hz, CH_2_OCH*CH_2_*-), 5.33 (s, 2H, -*CH_2_*-pyrazole), 7.43 (dd, 1H, *J* = 2.0 and 8.8 Hz, H7), 7.50 (d, 1H, *J* = 8.8 Hz, H8), 8.31 (d, 1H, *J* = 2.0 Hz, H5), 8.42 (s, 1H, H2); ^13^C-NMR (CDCl_3_, 100 MHz, *δ* ppm) 10.4, 12.3, 14.4, 45.2, 49.6, 53.1, 54.8, 60.9, 94.8, 111.6, 116.7, 125.9, 128.8, 131.5, 133.9, 137.4, 138.8, 146.6, 149.4, 165.4, 173.8; ESI-MS: *m/z* 459.9, 461.9 [M+H]^+^.

*Ethyl 6-((3,5-Dimethyl-1H-pyrazol-1-yl)methyl)-1-(2-methoxy-2-oxoethyl)-4-oxo-1,4-dihydroquino-line-3-carboxylate* (**7a**). Yield: 53%; mp: 225–228 °C; ^1^H-NMR (CDCl_3_, *δ* ppm) 1.41 (t, 3H, *J* = 7.2 Hz, -CH_2_*CH_3_*), 2.16 (s, 3H, *CH_3_*-pyrazole), 2.24 (s, 3H, *CH_3_*-pyrazole), 3.79 (s, 3H, -O*CH_3_*), 4.42 (q, 2H, *J* = 7.0 Hz, -*CH_2_*CH_3_), 4.84 (s, 2H, -CO*CH_2_*-), 5.31 (s, 2H, -*CH_2_*-pyrazole), 5.86 (s, 1H, H-pyrazole), 7.15 (d, 1H, *J* = 8.8 Hz, H8), 7.40 (dd, 1H, *J* = 2.0 and 8.4 Hz, H7), 8.28 (d, 1H, *J* = 2.0 Hz, H5), 8.39 (s, 1H, H2).

*Ethyl 6-((4-Chloro-3,5-dimethyl-1H-pyrazol-1-yl)methyl)-1-(2-methoxy-2-oxoethyl)-4-oxo-1,4-dihydro quinoline-3-carboxylate* (**7b**). Yield: 43%; mp: 235–237 °C; ^1^H-NMR (CDCl_3_, *δ* ppm) 1.43 (t, 3H, *J* = 7.2 Hz, -CH_2_*CH_3_*), 2.14 (s, 3H, *CH_3_*-pyrazole), 2.26 (s, 3H, *CH_3_*-pyrazole), 3.80 (s, 3H, -O*CH_3_*), 4.42 (q, 2H, *J* = 7.2 Hz, -*CH_2_*CH_3_), 4.84 (s, 2H, -CO*CH_2_*-), 5.31 (s, 2H, -*CH_2_*-pyrazole), 7.16 (d, 1H, *J* = 8.4 Hz, H8), 7.40 (dd, 1H, *J* = 2.4 and 8.8 Hz, H7), 8.32 (d, 1H, *J* = 2.0 Hz, H5), 8.39 (s, 1H, H2).

*Ethyl 6-((4-Bromo-3,5-dimethyl-1H-pyrazol-1-yl)methyl)-1-(2-methoxy-2-oxoethyl)-4-oxo-1,4-dihydro quinoline-3-carboxylate* (**7c**). Yield: 55%; mp: 239–242 °C; ^1^H-NMR (CDCl_3_, *δ* ppm) 1.43 (t, 3H, *J* = 7.2 Hz, -CH_2_*CH_3_*), 2.16 (s, 3H, *CH_3_*-pyrazole), 2.26 (s, 3H, *CH_3_*-pyrazole), 3.80 (s, 3H, -O*CH_3_*), 4.42 (q, 2H, *J* = 7.2 Hz, -*CH_2_*CH_3_), 4.84 (s, 2H, -CO*CH_2_*-), 5.34 (s, 2H, -*CH_2_*-pyrazole), 7.17 (d, 1H, *J* = 8.8 Hz, H8), 7.40 (dd, 1H, *J* = 2.0 and 8.4 Hz, H7), 8.33 (d, 1H, *J* = 2.0 Hz, H5), 8.41 (s, 1H, H2); ESI-MS: *m/z* 476, 478 [M+H]^+^, 498, 500 [M+Na]^+^, 514, 516 [M+K]^+^.

*Ethyl1-(3-(Acetylthio)propyl)-6-((3,5-dimethyl-1H-pyrazol-1-yl)methyl)-4-oxo-1,4-dihydroquinoline-3-carboxylate* (**8a**). Yield: 49%; mp: 125–128 °C; ^1^H-NMR (CDCl_3_, *δ* ppm) 1.43 (t, 3H, *J* = 7.2 Hz, -CH_2_*CH_3_*), 2.11–2.14 (m, 2H, -CH_2_*CH_2_*CH_2_S-), 2.16 (s, 3H, *CH_3_*-pyrazole), 2.24 (s, 3H, *CH_3_*-pyrazole), 2.38 (s, 3H, -CO*CH_3_*), 2.95 (t, 2H, *J* = 6.4 Hz, -CH_2_CH_2_*CH_2_*S-), 4.22 (t, 2H, *J* = 7.6 Hz, -*CH_2_*CH_2_CH_2_S-), 4.42 (q, 2H, *J* = 7.0 Hz, -*CH_2_*CH_3_), 5.31 (s, 2H, -*CH_2_*-pyrazole), 5.85 (s, 1H, *H*-pyrazole), 7.37–7.44 (m, 2H, H7 H8), 8.32 (s, 1H, H5), 8.47 (d, 1H, *J* = 8.4 Hz, H2); ^13^C-NMR (CDCl_3_, *δ* ppm) 11.1, 13.5, 14.4, 25.7, 28.9, 30.6, 51.9, 52.4, 60.9, 105.8, 111.4, 116.2, 126.1, 129.2, 131.5, 134.7, 138.1, 139.3, 147.9, 148.8, 165.7, 173.9, 194.9; ESI-MS: *m/z* 441.9 [M+H]^+^, 463.9 [M+Na]^+^, 479.8 [M+K]^+^.

*Ethyl 1-(3-(Acetylthio)propyl)-6-((4-chloro-3,5-dimethyl-1H-pyrazol-1-yl)methyl)-4-oxo-1,4-dihydro-quinoline-3-carboxylate* (**8b**). Yield: 51%; mp: 122–125 °C; ^1^H-NMR (CDCl_3_, *δ* ppm) 1.43 (t, 3H, *J* = 7.2 Hz, -CH_2_*CH_3_*), 2.13–2.17 (m, 2H, -CH_2_*CH_2_*CH_2_S-), 2.19 (s, 3H, *CH_3_*-pyrazole), 2.21 (s, 3H, *CH_3_*-pyrazole), 2.32 (s, 3H, -CO*CH_3_*), 2.96 (t, 2H, *J* = 6.8 Hz, -CH_2_CH_2_*CH_2_*S-), 4.22 (t, 2H, *J* = 7.6 Hz, -*CH_2_*CH_2_CH_2_S-), 4.44 (q, 2H, *J* = 7.2 Hz, -*CH_2_*CH_3_), 5.32 (s, 2H, -*CH_2_*-pyrazole), 7.39–7.46 (m, 2H, H7 H8), 8.35 (d, 1H, *J* = 2.0 Hz, H5), 8.45 (s, 1H, H2).

*Ethyl 1-(3-(Acetylthio)propyl)-6-((4-bromo-3,5-dimethyl-1H-pyrazol-1-yl)methyl)-4-oxo-1,4-dihydro- quinoline-3-carboxylate* (**8c**). Yield: 46%; mp: 141–143 °C; ^1^H-NMR (CDCl_3_, *δ* ppm) 1.43 (t, 3H, *J* = 7.2 Hz, -CH_2_*CH_3_*), 2.16 (s, 3H, *CH_3_*-pyrazole), 2.17–2.18 (m, 2H, -CH_2_*CH_2_*CH_2_S-), 2.24 (s, 3H, *CH_3_*-pyrazole), 2.38 (s, 3H, -CO*CH_3_*), 2.96 (t, 2H, *J* = 6.8 Hz, -CH_2_CH_2_*CH_2_*S-), 4.22 (t, 2H, *J* = 7.6 Hz, -*CH_2_*CH_2_CH_2_S-), 4.43 (q, 2H, *J* = 7.2 Hz, -*CH_2_*CH_3_), 5.34 (s, 2H, -*CH_2_*-pyrazole), 7.38–7.42 (m, 2H, H7 H8), 8.35 (d, 1H, *J* = 1.6 Hz, H5), 8.46 (s, 1H, H2).

*Ethyl 6-((3,5-Dimethyl-1H-pyrazol-1-yl)methyl)-1-(4-fluorobenzyl)-4-oxo-1,4-dihydroquinoline-3-carboxylate* (**9a**). Yield: 50%; mp: 209–211 °C; ^1^H-NMR (CDCl_3_, *δ* ppm) 1.43 (t, 3H, *J* = 7.2 Hz, -CH_2_*CH_3_*), 2.14 (s, 3H, *CH_3_*-pyrazole), 2.21 (s, 3H, *CH_3_*-pyrazole), 4.42 (q, 2H, *J* = 7.0 Hz, -*CH_2_*CH_3_), 5.28 (s, 2H, -*CH_2_*-pyrazole), 5.33 (s, 2H, -*CH_2_*-Ar), 5.83 (s, 1H, *H*-pyrazole), 7.05 (t, 2H, *J* = 6.4 Hz, Ar-*H*), 7.13 (q, 2H, *J* = 8.8 Hz, Ar-*H*), 7.24 (d, 1H, *J* = 8.8 Hz, H8), 7.29 (dd, 1H, *J* = 2.0 and 8.8 Hz, H7), 8.30 (d, 1H, *J* = 2.0 Hz, H5), 8.57 (s, 1H, H2); ESI-MS: *m/z* 433.9 [M+H]^+^, 455.9 [M+Na]^+^, 471.8 [M+K]^+^.

*Ethyl 6-((4-Chloro-3,5-dimethyl-1H-pyrazol-1-yl)methyl)-1-(4-fluorobenzyl)-4-oxo-1,4-dihydroquinoline-3-carboxylate* (**9b**). Yield: 51%; mp: 226–228 °C; ^1^H-NMR (CDCl_3_, *δ* ppm) 1.44 (t, 3H, *J* = 7.2 Hz, -CH_2_*CH_3_*), 2.13 (s, 3H, *CH_3_*-pyrazole), 2.20 (s, 3H, *CH_3_*-pyrazole), 4.42 (q, 2H, *J* = 7.2 Hz, -*CH_2_*CH_3_), 5.28 (s, 2H, -*CH_2_*-pyrazole), 5.33 (s, 2H, -*CH_2_*-Ar), 7.05 (t, 2H, *J* = 6.8 Hz, Ar-*H*), 7.07 (q, 2H, *J* = 8.0 Hz, Ar-*H*), 7.27 (d, 1H, *J* = 8.8 Hz, H8), 7.30 (dd, 1H, *J* = 2.4 and 8.8 Hz, H7), 8.32 (d, 1H, *J* = 1.6 Hz, H5), 8.58 (s, 1H, H2); ESI-MS: *m/z* 467.9 [M+H]^+^.

*Ethyl 6-((4-Bromo-3,5-dimethyl-1H-pyrazol-1-yl)methyl)-1-(4-fluorobenzyl)-4-oxo-1,4-dihydroquinoline-3-carboxylate* (**9c**). Yield: 55%; mp: 219–221 °C; ^1^H-NMR (CDCl_3_, *δ* ppm) 1.44 (t, 3H, *J* = 7.2 Hz, -CH_2_*CH_3_*), 2.16 (s, 3H, *CH_3_*-pyrazole), 2.22 (s, 3H, *CH_3_*-pyrazole), 4.42 (q, 2H, *J* = 7.0 Hz, -*CH_2_*CH_3_), 5.32 (s, 2H, -*CH_2_*-pyrazole), 5.35 (s, 2H, -*CH_2_*-Ar), 7.06 (t, 2H, *J* = 8.4 Hz, Ar-*H*), 7.16 (q, 2H, *J* = 9.2 Hz, Ar-*H*), 7.27 (d, 1H, *J* = 8.8 Hz, H8), 7.31 (dd, 1H, *J* = 2.0 and 8.8 Hz, H7), 8.30 (d, 1H,*J* = 1.2 Hz, H5), 8.59 (s, 1H, H2); ESI-MS: *m/z* 521, 514 [M+H]^+^, 534, 536 [M+Na]^+^, 550, 552 [M+K]^+^.

*Ethyl 6-((3,5-Dimethyl-1H-pyrazol-1-yl)methyl)-1-(4-nitrobenzyl)-4-oxo-1,4-dihydroquinoline-3-carboxylate* (**10a**). Yield: 49%; mp: 194–196 °C; ^1^H-NMR (CDCl_3_, *δ* ppm) 1.43 (t, 3H, *J* = 7.2 Hz, -CH_2_*CH_3_*), 2.15 (s, 3H, *CH_3_*-pyrazole), 2.19 (s, 3H, *CH_3_*-pyrazole), 4.42 (q, 2H, *J* = 7.2 Hz, -*CH_2_*CH_3_), 5.27 (s, 2H, -*CH_2_*-pyrazole), 5.47 (s, 2H, -*CH_2_*-Ar), 5.83 (s, 1H, *H*-pyrazole), 7.10 (d, 1H, *J* = 8.8 Hz, H8), 7.24 (dd, 1H, *J* = 2.4 and 8.8 Hz, H7), 7.31 (d, 2H, *J* = 8.8 Hz, Ar-*H*), 8.22 (d, 2H, *J* = 6.0 Hz, Ar-*H*), 8.27 (d, 1H, *J* = 2.0 Hz, H5), 8.56 (s, 1H, H2).

*Ethyl 6-((4-Chloro-3,5-dimethyl-1H-pyrazol-1-yl)methyl)-1-(4-nitrobenzyl)-4-oxo-1,4-dihydroquinoline-3-carboxylate* (**10b**). Yield: 39%; mp: 232–235 °C; ^1^H-NMR (CDCl_3_, *δ* ppm) 1.44 (t, 3H, *J* = 7.2 Hz, -CH_2_*CH_3_*), 2.13 (s, 3H, *CH_3_*-pyrazole), 2.19 (s, 3H, *CH_3_*-pyrazole), 4.42 (q, 2H, *J* = 7.0 Hz, -*CH_2_*CH_3_), 5.27 (s, 2H, -*CH_2_*-pyrazole), 5.47 (s, 2H, -*CH_2_*-Ar), 7.12 (d, 1H, *J* = 8.4 Hz, H8), 7.28 (dd, 1H, *J* = 2.0 and 8.4 Hz, H7), 7.32 (d, 2H, *J* = 8.8 Hz, Ar-*H*), 8.23 (d, 2H, *J* = 6.8 Hz, Ar-*H*), 8.33 (d, 1H, *J* = 2.0 Hz, H5), 8.58 (s, 1H, H2); ESI-MS: *m/z* 516.8 [M+Na]^+^, 493.0 [M-H]^−^.

*Ethyl 6-((4-Bromo-3,5-dimethyl-1H-pyrazol-1-yl)methyl)-1-(4-nitrobenzyl)-4-oxo-1,4-dihydroquinoline-3-carboxylate* (**10c**). Yield: 40%; mp: 240–242 °C; ^1^H-NMR (CDCl_3_, *δ* ppm) 1.43 (t, 3H, *J* = 7.2 Hz, -CH_2_*CH_3_*), 2.15 (s, 3H, *CH_3_*-pyrazole), 2.19 (s, 3H, *CH_3_*-pyrazole), 4.42 (q, 2H, *J* = 7.0 Hz, -*CH_2_*CH_3_), 5.30 (s, 2H, -*CH_2_*-pyrazole), 5.47 (s, 2H, -*CH_2_*-Ar), 7.12 (d, 1H, *J* = 8.8 Hz, H8), 7.28 (dd, 1H, *J* = 2.0 and 8.8 Hz, H7), 7.32 (d, 2H, *J* = 8.8 Hz, Ar-*H*), 8.23 (d, 2H, *J* = 6.4 Hz, Ar-*H*), 8.33 (d, 1H, *J* = 1.6 Hz, H5), 8.58 (s, 1H, H2).

### 3.3. General Procedure for the Synthesis of 1-Substitued-6-(pyrazolylmethyl)-4-oxo-4H-quinoline-3-carboxylic Acids ***11**–**16***

A suspension of the appropriate ester **5**–**8** (0.5 mmol) in 10% aq. NaOH (10 mL) was refluxed for 2 h. After cooling at room temperature, the reaction mixture was acidified using conc. HCl. The resulting precipitate was filtered and washed with water and DCM to give the corresponding 4-quinolone-3-carboxylic acids **11–14**. Alternatively, a suspension of the appropriate ester **9–10** (0.5 mmol) in 10% aq. HCl (10 mL) was refluxed for 2 h. After cooling at room temperature, the resulting precipitate was filtered and washed with water and DCM to give the corresponding 4-quinolone-3-carboxylic acids **15–16**.

*6-((3,5-Dimethyl-1H-pyrazol-1-yl)methyl)-4-oxo-1,4-dihydroquinoline-3-carboxylic acid* (**11a**). Yield: 63%; mp: 254–256 °C; ^1^H-NMR (DMSO-*d_6_*, *δ* ppm) 2.11 (s, 3H, *CH_3_*-pyrazole), 2.18 (s, 3H, *CH_3_*-pyrazole), 5.38 (s, 2H, -*CH_2_*-pyrazole), 5.89 (s, 1H, H-pyrazole), 7.64 (dd, 1H, *J* = 2.0 and 8.8 Hz, H7), 7.81 (d, 1H, *J* = 8.4 Hz, H8), 7.99 (d, 1H, *J* = 1.6 Hz, H5), 8.84 (s, 1H, H2), 13.36 (s, 1H, -*NH*-), 15.29 (s, 1H, -COO*H*); ^13^C-NMR (DMSO-*d_6_*, *δ* ppm) 11.1, 13.8, 51.5, 105.7, 108.2, 120.9, 123.2, 124.8, 133.2, 136.6, 139.4, 139.5, 145.8, 146.9, 166.9, 178.4; HRMS: *m/z* calcd for C_16_H_16_N_3_O_3_: 298.1186; found: 298.1188; HPLC purity 98.36%.

*6-((4-Chloro-3,5-dimethyl-1H-pyrazol-1-yl)methyl)-4-oxo-1,4-dihydroquinoline-3-carboxylic acid* (**11b**). Yield: 47%; mp: 263–265 °C; ^1^H-NMR (DMSO-*d_6_*, *δ* ppm) 2.09 (s, 3H, *CH_3_*-pyrazole), 2.19 (s, 3H, *CH_3_*-pyrazole), 5.45 (s, 2H, -*CH_2_*-pyrazole), 7.66 (dd, 1H, *J* = 2.0 and 8.4 Hz, H7), 7.82 (d, 1H, *J* = 8.4 Hz, H8), 8.04 (d, 1H, *J* = 1.6 Hz, H5), 8.88 (s, 1H, H2), 13.51 (s, 1H, -*NH*-), 15.29 (s, 1H, -COO*H*); ^13^C-NMR (DMSO-*d_6_*, *δ* ppm) 9.5, 11.5, 52.7, 107.0, 108.18, 120.9, 123.5, 124.8, 133.3, 135.7, 136.1, 139.4, 144.0, 145.7, 166.8, 178.5; HRMS: *m/z* calcd for C_16_H_15_ClN_3_O_3_: 332.0796; found: 332.0799; HPLC purity 99.40%.

*6-((4-Bromo-3,5-dimethyl-1H-pyrazol-1-yl)methyl)-4-oxo-1,4-dihydroquinoline-3-carboxylic acid* (**11c**). Yield: 81%; mp: 253–256 °C; ^1^H-NMR (DMSO-*d_6_*, *δ* ppm) 2.09 (s, 3H, *CH_3_*-pyrazole), 2.17 (s, 3H, *CH_3_*-pyrazole), 5.44 (s, 2H, -*CH_2_*-pyrazole), 7.64 (dd, 1H, *J* = 2.0 and 8.4 Hz, H7), 7.79 (d, 1H, *J* = 8.4 Hz, H8), 8.01 (d, 1H, *J* = 1.6 Hz, H5), 8.84 (s, 1H, H2), 13.36 (s, 1H, -*NH*-), 15.20 (s, 1H, -COO*H*); ^13^C-NMR (DMSO-*d_6_*, *δ* ppm) 10.4, 12.5, 52.8, 93.8, 108.2, 120.8, 123.6, 124.8, 133.4, 135.8, 137.8, 139.3, 145.5, 145.6, 166.7, 178.5; HRMS: *m/z* calcd for C_16_H_15_BrN_3_O_3_: 376.0291, 378.0276; found: 376.0296, 378.0278; HPLC purity 97.17%.

*1-(2,3-Dihydroxypropyl)-6-((3,5-dimethyl-1H-pyrazol-1-yl)methyl)-4-oxo-1,4-dihydroquinoline-3-carboxylic acid*(**12a**). Yield: 52%; mp: 187–189 °C; ^1^H-NMR (DMSO-*d_6_*, *δ* ppm) 2.19 (s, 3H, *CH_3_*-pyrazole), 2.27 (s, 3H, *CH_3_*-pyrazole), 3.40–3.55 (m, 2H, HOCH_2_CHOH*CH_2_*-), 3.81–3.83 (m, 1H, HOCH_2_*CH*OHCH_2_-), 4.28 (q, 1H, *J* = 14.4 Hz, HO*CH_2_*CHOHCH_2_-), 4.82 (dd, 1H, *J* = 2.4 and 10.4 Hz, HO*CH_2_*CHOHCH_2_-), 5.53 (s, 2H, -*CH_2_*-pyrazole), 6.08 (s, 1H, *H*-pyrazole), 6.31 (br, 2H, *HO*CH_2_CH*OH*CH_2_-), 7.75 (dd, 1H, *J* = 2.0 and 9.2 Hz, H7), 8.02 (d, 1H, *J* = 9.2 Hz, H8), 8.12 (d, 1H, *J* = 2.0 Hz, H5), 8.86 (s, 1H, H2), 15.01 (s, 1H, -COO*H*); ^13^C-NMR (DMSO-*d_6_*, *δ* ppm) 11.1, 13.2, 51.1, 57.4, 63.7, 69.5, 106.5, 107.7, 119.3, 124.3, 125.9, 133.4, 135.6, 139.5, 141.3, 146.8, 151.0, 166.5, 177.9; HRMS: *m/z* calcd for C_19_H_22_N_3_O_5_: 372.1554; found: 372.1556; HPLC purity 98.10%.

*1-(2,3-Dihydroxypropyl)-6-((4-chloro-3,5-dimethyl-1H-pyrazol-1-yl)methyl)-4-oxo-1,4-dihydro-quinoline-3-carboxylic acid* (**12b**). Yield: 63%; mp: 278–280 °C; ^1^H-NMR (DMSO-*d_6_*, *δ* ppm) 2.12 (s, 3H, *CH_3_*-pyrazole), 2.20 (s, 3H, *CH_3_*-pyrazole), 3.39–3.55 (m, 2H, HOCH_2_CHOH*CH_2_*-), 3.82 (m, 1H, HOCH_2_*CH*OHCH_2_-), 4.27 (q, 1H, *J* = 14.4 Hz, HO*CH_2_*CHOHCH_2_-), 4.80 (dd, 1H, *J* = 2.0 and 14.4 Hz, HO*CH_2_*CHOHCH_2_-), 4.96 (t, 1H, *J* = 5.6 Hz, *HO*CH_2_CHOHCH_2_-), 5.22 (d, 1H, *J* = 5.6 Hz, HOCH_2_CH*OH*CH_2_-), 5.48 (s, 2H, -*CH_2_*-pyrazole), 7.73 (dd, 1H, *J* = 2.0 and 8.8 Hz, H7), 8.00 (d, 1H, *J* = 9.2 Hz, H8), 8.13 (d, 1H, *J* = 1.6 Hz, H5), 8.86 (s, 1H, H2), 15.13 (s, 1H, -COO*H*); ^13^C-NMR (DMSO-*d_6_*, *δ* ppm) 9.5, 11.5, 52.4, 57.5, 63.8, 69.4, 107.5, 107.7, 119.2, 124.4, 125.9, 133.4, 135.8, 136.1, 139.5, 144.1, 151.0, 166.5, 178.0; HRMS: *m/z* calcd for C_19_H_21_ClN_3_O_5_: 406.1164; found: 406.1167; HPLC purity 97.%.

*1-(2,3-Dihydroxypropyl)-6-((4-bromo-3,5-dimethyl-1H-pyrazol-1-yl)methyl)-4-oxo-1,4-dihydroquino-line-3-carboxylic acid* (**12c**). Yield: 62%; mp: 267–269 °C; ^1^H-NMR (DMSO-*d_6_*, *δ* ppm) 2.19 (s, 3H, *CH_3_*-pyrazole), 2.27 (s, 3H, *CH_3_*-pyrazole), 3.39–3.53 (m, 2H, HOCH_2_CHOH*CH_2_*-), 3.81 (m, 1H, HOCH_2_*CH*OHCH_2_-), 4.27 (q, 1H, *J* = 10.8 Hz, HO*CH_2_*CHOHCH_2_-), 4.80 (d, 1H, *J* = 12.8 Hz, HO*CH_2_*CHOHCH_2_-), 4.98 (t, 1H, *J* = 5.6 Hz, *HO*CH_2_CHOHCH_2_-), 5.22 (d, 1H, *J* = 5.6 Hz, HOCH_2_CH*OH*CH_2_-), 5.51 (s, 2H, -*CH_2_*-pyrazole), 7.73 (dd, 1H, *J* = 1.6 and 8.8 Hz, H7), 8.00 (d, 1H, *J* = 4.8 Hz, H8), 8.13 (s, 1H, H5), 8.86 (s, 1H, H2), 15.15 (s, 1H, -COO*H*); ^13^C-NMR (DMSO-*d_6_*, *δ* ppm) 10.4, 12.5, 52.5, 57.4, 63.7, 69.4, 93.8, 107.7, 119.2, 124.4, 125.9, 133.4, 135.8, 137.8, 139.5, 145.6, 151.0, 166.5, 178.0; HRMS: *m/z* calcd for C_19_H_21_BrN_3_O_5_: 450.0659, 452.0644; found: 450.0664, 452.0651; HPLC purity 97.73%.

*1-(Carboxymethyl)-6-((3,5-dimethyl-1H-pyrazol-1-yl)methyl)-4-oxo-1,4-dihydroquinoline-3-carboxylic acid* (**13a**). Yield: 78%; mp > 300 °C; ^1^H-NMR (DMSO-*d_6_*, *δ* ppm) 2.09 (s, 3H, *CH_3_*-pyrazole), 2.17 (s, 3H, *CH_3_*-pyrazole), 5.38 (s, 2H, -*CH_2_*-pyrazole), 5.42 (s, 2H, -*CH_2_*COOH), 5.88 (s, 1H, H-pyrazole), 7.68 (dd, 1H, *J* = 2.0 and 8.8 Hz, H7), 7.79 (d, 1H, *J* = 8.8 Hz, H8), 8.05 (d, 1H, *J* = 1.6 Hz, H5), 9.02 (s, 1H, H2), 13.63 (s, 1H, -*NH*-), 14.97 (s, 1H, -COO*H*); ^13^C-NMR (DMSO-*d_6_*, *δ* ppm) 11.1, 13.8, 51.2, 54.7, 105.7, 108.3, 118.8, 124.0, 125.5, 133.6, 136.9, 139.4, 139.7, 147.0, 151.1, 166.2, 169.2, 178.2; HRMS: *m/z* calcd for C_18_H_18_N_3_O_5_: 356.1241; found: 356.1242; HPLC purity 97.80%.

*1-(Carboxymethyl)-6-((4-chloro-3,5-dimethyl-1H-pyrazol-1-yl)methyl)-4-oxo-1,4-dihydroquinoline-3-carboxylic acid* (**13b**). Yield: 58%; mp: 292–295 °C; ^1^H-NMR (DMSO-*d_6_*, *δ* ppm) 2.12 (s, 3H, *CH_3_*-pyrazole), 2.20 (s, 3H, *CH_3_*-pyrazole), 5.35 (s, 2H, -*CH_2_*-pyrazole), 5.46 (s, 2H, -*CH_2_*COOH), 7.71 (dd, 1H, *J* = 2.0 and 8.8 Hz, H7), 7.79 (d, 1H, *J* = 9.2 Hz, H8), 8.12 (d, 1H, *J* = 1.6 Hz, H5), 9.07 (s, 1H, H2), 15.01 (s, 1H, -COO*H*); ^13^C-NMR (DMSO-*d_6_*, *δ* ppm) 9.5, 11.5, 52.3, 55.4, 107.0, 108.2, 119.1, 124.2, 125.5, 133.7, 135.9, 136.2, 139.9, 144.1, 151.1, 166.3, 169.1, 178.1; HRMS: *m/z* calcd for C_18_H_17_ClN_3_O_5_: 390.0851; found: 390.0855; HPLC purity 99.15%.

*1-(Carboxymethyl)-6-((4-bromo-3,5-dimethyl-1H-pyrazol-1-yl)methyl)-4-oxo-1,4-dihydro-quinoline-3-carboxylic acid* (**13c**). Yield: 72%; mp: 278–280 °C; ^1^H-NMR (DMSO-*d_6_*, *δ* ppm) 2.12 (s, 3H, *CH_3_*-pyrazole), 2.21 (s, 3H, *CH_3_*-pyrazole), 5.36 (s, 2H, -*CH_2_*-pyrazole), 5.48 (s, 2H, -*CH_2_*COOH), 7.71 (dd, 1H, *J* = 2.0 and 9.2 Hz, H7), 7.79 (d, 1H, *J* = 9.2 Hz, H8), 8.12 (d, 1H, *J* = 2.0 Hz, H5), 9.07 (s, 1H, H2), 14.99 (s, 1H, -COO*H*); ^13^C-NMR (DMSO-*d_6_*, *δ* ppm) 10.4, 12.5, 52.5, 55.3, 93.8, 108.3, 119.0, 124.3, 125.6, 133.7, 135.9, 137.8, 139.9, 145.6, 151.1, 166.2, 169.1, 178.2; HRMS: *m/z* calcd for C_18_H_17_BrN_3_O_5_: 434.0346, 436.0331; found: 434.0350, 436.0329; HPLC purity 99.23%.

*1-(3-Mercaptopropyl)-6-((3,5-dimethyl-1H-pyrazol-1-yl)methyl)-4-oxo-1,4-dihydroquinoline-3-carboxylic acid* (**14a**). Yield: 65%; mp: 225–226 °C; ^1^H-NMR (DMSO-*d_6_*, *δ* ppm) 2.08 (m, 2H, -CH_2_*CH_2_*CH_2_SH), 2.12 (s, 3H, *CH_3_*-pyrazole), 2.19 (s, 3H, *CH_3_*-pyrazole), 2.54–2.58 (m, 2H, -CH_2_CH_2_*CH_2_*SH), 4.62 (t, 2H, *J* = 7.2 Hz, -*CH_2_*CH_2_CH_2_SH), 5.41 (s, 2H, -*CH_2_*-pyrazole), 5.89 (s, 1H, *H*-pyrazole), 7.71 (dd, 1H, *J* = 2.0 and 8.8 Hz, H7), 8.05 (d, 1H, *J* = 8.8 Hz, H8), 8.08 (d, 1H, *J* = 2.0 Hz, H5), 9.02 (s, 1H, H2), 15.13 (s, 1H, -COO*H*); ^13^C-NMR (DMSO-*d_6_*, 100 MHz, *δ* ppm) 11.1, 13.8, 21.1, 28.5, 34.3, 51.2, 105.7, 108.2, 118.9, 124.2, 126.1, 133.5, 136.8, 138.9, 139.5, 147.0, 149.9, 166.5, 178.0; HRMS: *m/z* calcd for C_19_H_22_N_3_O_3_S: 372.1376; found: 372.1380; HPLC purity 97.50%.

*1-(3-Mercaptopropyl)-6-((4-chloro-3,5-dimethyl-1H-pyrazol-1-yl)methyl)-4-oxo-1,4-dihydro-quinoline-3-carboxylic acid* (**14b**). Yield: 54%; mp: 238–240 °C; ^1^H-NMR (DMSO-*d_6_*, *δ* ppm) 2.08–2.09 (m, 2H, -CH_2_*CH_2_*CH_2_SH), 2.11 (s, 3H, *CH_3_*-pyrazole), 2.20 (s, 3H, *CH_3_*-pyrazole), 2.56–2.58 (m, 2H, -CH_2_CH_2_*CH_2_*SH), 4.62 (t, 2H, *J* = 7.2 Hz, -*CH_2_*CH_2_CH_2_SH), 5.45 (s, 2H, -*CH_2_*-pyrazole), 7.73 (dd, 1H, *J* = 2.0 and 8.8 Hz, H7), 8.06 (d, 1H, *J* = 8.4 Hz, H8), 8.14 (d, 1H, *J* = 2.0 Hz, H5), 9.03 (s, 1H, H2), 15.09 (s, 1H, -COO*H*); ^13^C-NMR (DMSO-*d_6_*, *δ* ppm) 9.5, 11.5, 21.1, 28.6, 34.3, 52.4, 107.1, 108.3, 119.0, 124.5, 126.1, 133.6, 135.9, 136.1, 139.1, 144.1, 149.9, 166.4, 178.0; HRMS: *m/z* calcd for C_19_H_21_ClN_3_O_3_S: 406.0986; found: 406.0989; HPLC purity 96.68%.

*6-((4-Bromo-3,5-dimethyl-1H-pyrazol-1-yl)methyl)-1-(3-mercaptopropyl)-4-oxo-1,4-dihydroquinoline-3-carboxylic acid* (**14c**). Yield: 42%; mp: 231–233 °C; ^1^H-NMR (DMSO-*d_6_*, *δ* ppm) 2.08–2.11 (m, 2H, -CH_2_*CH_2_*CH_2_SH), 2.12 (s, 3H, *CH_3_*-pyrazole), 2.21 (s, 3H, *CH_3_*-pyrazole), 2.55–2.58 (m, 2H, -CH_2_CH_2_*CH_2_*SH), 4.62 (t, 2H, *J* = 7.2 Hz, -*CH_2_*CH_2_CH_2_SH), 5.51 (s, 2H, -*CH_2_*-pyrazole), 7.73 (dd, 1H, *J* = 2.0 and 8.8 Hz, H7), 8.06 (d, 1H, *J* = 8.8 Hz, H8), 8.14 (d, 1H, *J* = 2.0 Hz, H5), 9.03 (s, 1H, H2), 15.09 (s, 1H, -COO*H*); ^13^C-NMR (DMSO-*d_6_*, *δ* ppm) 10.4, 12.5, 21.1, 28.4, 34.3, 52.5, 93.9, 108.3, 119.1, 124.5, 126.1, 133.6, 135.9, 137.8, 139.0, 145.6, 149.9, 166.4, 178.0; HRMS: *m/z* calcd for C_19_H_21_BrN_3_O_3_S: 450.0481, 452.0466; found: 450.0489, 452.0467; HPLC purity 96.90%.

*1-(4-Fluorobenzyl)-6-((3,5-dimethyl-1H-pyrazol-1-yl)methyl)-4-oxo-1,4-dihydro-quinoline-3-carboxylic acid*(**15a**). Yield: 63%; mp: 271–273 °C; ^1^H-NMR (DMSO-*d_6_*, *δ* ppm) 2.09 (s, 3H, *CH_3_*-pyrazole), 2.16 (s, 3H, *CH_3_*-pyrazole), 5.36 (s, 2H, -*CH_2_*-pyrazole), 5.82 (s, 2H, -*CH_2_*-Ar), 5.87 (s, 1H, *H*-pyrazole), 7.19 (t, 2H, *J* = 8.8 Hz, Ar-*H*), 7.37 (q, 2H, *J* = 8.4 Hz, Ar-*H*), 7.60 (dd, 1H, *J* = 2.0 and 9.2 Hz, H7), 7.87 (d, 1H, *J* = 8.8 Hz, H8), 8.05 (d, 1H, *J* = 1.6 Hz, H5), 9.26 (s, 1H, H2), 15.06 (s, 1H, -COO*H*); ^13^C-NMR (DMSO-*d_6_*, *δ* ppm) 11.1, 13.8, 51.1, 56.2, 105.7, 108.5, 116.1, 116.4, 119.5, 124.1, 126.2, 129.5, 129.5, 133.4, 136.9, 139.1, 139.4, 147.0, 150.5, 166.4, 178.2; HRMS: *m/z* calcd for C_23_H_21_FN_3_O_3_: 406.1561; found: 406.1569; HPLC purity 99.70%.

*1-(4-Fluorobenzyl)-6-((4-chloro-3,5-dimethyl-1H-pyrazol-1-yl)methyl)-4-oxo-1,4-dihydro-quinoline-3-carboxylic acid* (**15b**). Yield: 75%; mp: 239–241 °C; ^1^H-NMR (DMSO-*d_6_*, *δ* ppm) 2.17 (s, 3H, *CH_3_*-pyrazole), 2.22 (s, 3H, *CH_3_*-pyrazole), 5.34 (s, 2H, -*CH_2_*-pyrazole), 5.49 (s, 2H, -*CH_2_*-Ar), 7.10 (t, 2H, *J* = 8.0 Hz, Ar-*H*), 7.14–7.18 (m, 2H, Ar-*H*), 7.42–7.49 (m, 2H, H7 H8), 8.35 (s, 1H, H5), 8.89 (s, 1H, H2), 14.68 (s, 1H, -COO*H*); ^13^C-NMR (DMSO-*d_6_*, 100 MHz, *δ* ppm) 9.5, 11.4, 52.7, 57.6, 108.7, 109.4, 116.6, 116.8, 118.1, 125.1, 126.7, 128.0, 128.1, 132.8, 135.4, 135.6, 139.0, 145.4, 149.1, 166.6, 178.3; HRMS: *m/z* calcd for C_23_H_20_ClFN_3_O_3_: 440.1171; found: 440.1169; HPLC purity 99.55%.

*1-(4-Fluorobenzyl)-6-((4-bromo-3,5-dimethyl-1H-pyrazol-1-yl)methyl)-4-oxo-1,4-dihydro-quinoline-3-carboxylic acid* (**15c**). Yield: 73%; mp: 255–257 °C; ^1^H-NMR (DMSO-*d_6_*, *δ* ppm) 2.17 (s, 3H, *CH_3_*-pyrazole), 2.21 (s, 3H, *CH_3_*-pyrazole), 5.35 (s, 2H, -*CH_2_*-pyrazole), 5.48 (s, 2H, -*CH_2_*-Ar), 7.08 (t, 2H, *J* = 7.6 Hz, Ar-*H*), 7.14 (s, 2H, Ar-*H*), 7.43 (s, 2H, H7 H8), 8.35 (s, 1H, H5), 8.88 (s, 1H, H2), 15.06 (s, 1H, -COO*H*); ^13^C-NMR (DMSO-*d_6_*, *δ* ppm) 10.4, 12.31, 52.8, 57.6, 109.4, 116.6, 116.8, 118.0, 125.2, 126.7, 128.0, 128.1, 132.8, 135.4, 137.4, 139.1, 139.0, 146.9, 149.1, 166.5, 178.3; HRMS: *m/z* calcd for C_23_H_20_BrFN_3_O_3_: 484.0666, 486.0651; found: 484.0665, 486.0646; HPLC purity 99.57%.

*1-(4-Nitrobenzyl)-6-((3,5-dimethyl-1H-pyrazol-1-yl)methyl)-4-oxo-1,4-dihydro-quinoline-3-carboxylic acid* (**16a**). Yield: 56%; mp: 274–276 °C; ^1^H-NMR (DMSO-*d_6_*, *δ* ppm) 2.08 (s, 3H, *CH_3_*-pyrazole), 2.16 (s, 3H, *CH_3_*-pyrazole), 5.36 (s, 2H, -*CH_2_*-pyrazole), 5.87 (s, 1H, *H*-pyrazole), 6.01 (s, 2H, -*CH_2_*-Ar), 7.52 (d, 2H, *J* = 8.8 Hz, Ar-*H*), 7.58 (dd, 1H, *J* = 2.0 and 8.8 Hz, H7), 7.75 (d, 1H, *J* = 8.8 Hz, H8), 8.07 (d, 1H, *J* = 2.0 Hz, H5), 8.18 (d, 2H, *J* = 8.8 Hz, Ar-*H*), 9.32 (s, 1H, H2), 15.01 (s, 1H, -COO*H*); ^13^C-NMR (DMSO-*d_6_*, *δ* ppm) 11.1, 13.8, 51.1, 56.3, 105.7, 108.8, 119.3, 124.2, 124.4, 126.2, 128.3, 133.6, 137.1, 139.0, 139.4, 143.5, 147.0, 147.6, 150.9, 166.3, 178.4; HRMS: *m/z* calcd for C_23_H_21_N_4_O_5_: 433.1506; found: 433.1508; HPLC purity 99.80%.

*1-(4-Nitrobenzyl)-6-((4-chloro-3,5-dimethyl-1H-pyrazol-1-yl)methyl)-4-oxo-1,4-dihydroquinoline-3-carboxylic acid* (**16b**). Yield: 55%; mp: 282–284 °C; ^1^H-NMR (DMSO-*d_6_*, *δ* ppm) 2.09 (s, 3H, *CH_3_*-pyrazole), 2.17 (s, 3H, *CH_3_*-pyrazole), 5.42 (s, 2H, -*CH_2_*-pyrazole), 6.01 (s, 2H, -*CH_2_*-Ar), 7.53 (d, 2H, *J* = 8.8 Hz, Ar-*H*), 7.60 (dd, 1H, *J* = 2.0 and 8.8 Hz, H7), 7.75 (d, 1H, *J* = 6.8 Hz, H8), 8.13 (d, 1H, *J* = 2.0 Hz, H5), 8.19 (d, 2H, *J* = 8.8 Hz, Ar-*H*), 9.33 (s, 1H, H2), 14.98 (s, 1H, -COO*H*); ^13^C-NMR (DMSO-*d_6_*, *δ* ppm) 9.5, 11.5, 52.2, 56.3, 107.7, 108.8, 119.5, 124.4, 124.5, 126.2, 128.3, 133.7, 136.2, 139.2, 143.5, 144.1, 147.6, 151.0, 166.3, 178.3; HRMS: *m/z* calcd for C_23_H_20_ClN_4_O_5_: 467.1116; found: 467.1124; HPLC purity 97.32%.

*1-(4-Nitrobenzyl)-6-((4-bromo-3,5-dimethyl-1H-pyrazol-1-yl)methyl)-4-oxo-1,4dihydroquinoline-3-carboxylic acid* (**16c**). Yield: 69%; mp: 266–268 °C; ^1^H-NMR (DMSO-*d_6_*, *δ* ppm) 2.09 (s, 3H, *CH_3_*-pyrazole), 2.18 (s, 3H, *CH_3_*-pyrazole), 5.45 (s, 2H, -*CH_2_*-pyrazole), 6.01 (s, 2H, -*CH_2_*-Ar), 7.53 (d, 2H, *J* = 8.8 Hz, Ar-*H*), 7.60 (dd, 1H, *J* = 2.0 and 8.8 Hz, H7), 7.75 (d, 1H, *J* = 8.8 Hz, H8), 8.13 (d, 1H, *J* = 1.6 Hz, H5), 8.19 (d, 2H, *J* = 8.8 Hz, Ar-*H*), 9.33 (s, 1H, H2), 14.99 (s, 1H, -COO*H*); ^13^C-NMR (DMSO-*d_6_*, *δ* ppm) 10.4, 12.5, 52.3, 56.4, 93.8, 108.8, 119.5, 124.4, 124.5, 126.6, 128.3, 133.7, 136.2, 137.9, 139.2, 143.5, 145.7, 147.6, 150.9, 166.3, 178.3; HRMS: *m/z* calcd for C_23_H_20_BrN_4_O_5_: 511.0611, 513.0596; found: 511.0616, 513.0599; HPLC purity 97.64%.

## 4. Conclusions

A series of 6-(pyrazolylmethyl)-4-oxo-4*H*-quinoline-3-carboxylic acid derivatives were synthesized to rationalize the influence on the anti-IN activity of the 4-oxo-4*H*-quinolizine-3-carboxylic acid scaffold of different substituents at N-1 and replacement of the phenyl group by a pyrazole ring at C-6, which allowed us to highlight new SAR aspects of quinoline IN inhibitors. However, the HIV-1 integrase strand transfer (ST) activity assay results showed no obvious inhibitory activities for compounds **11–16**. Therefore, the replacement of the benzene ring at C-6 by another bioisosteric moiety and an appropriate substituent at N-1 position are essential to enhance the structure-activity relationships study for new quinoline derivatives with potential anti-IN activity.

## References

[1] Tan J.J., Cong X.J., Hu L.M., Wang C.X., Jia L., Liang X.J. (2010). Therapeutic strategies underpinning the development of novel techniques for the treatment of HIV infection. Drug Discov. Today.

[2] Pommier Y., Johnson A.A., Marchand C. (2005). Integrase inhibitors to treat HIV/Aids. Nat. Rev. Drug Discov..

[3] Boros E.E., Johns B.A., Garvey E.P., Koble C.S., Miller W.H. (2006). Synthesis and HIV integrase strand transfer activity of 7-hydroxy[1,3] thiazolo [5,4-*b*] pyridin-5(4*H*)-ones. Bioorg. Med. Chem. Lett..

[4] Tabarrini O., Massari S., Daelemans D., Stevens M., Manfroni G., Sabatini S., Balzarini J., Cecchetti V., Pannecouque C., Fravolini A. (2008). Structure-activity relationship study on Anti-HIV 6-desfluoroquinolones. J. Med. Chem..

[5] Cecchetti V., Parolin C., Moro S., Pecere T., Filipponi E., Calistri A., Tabarrini O., Gatto B., Palumbo M., Fravolini A., Palu G. (2000). 6-Aminoquinolones as new potential anti-HIV agents. J. Med. Chem..

[6] Lampiris H.W. (2012). Elvitegravir: A once-daily, boosted, HIV-1 integrase inhibitor. Expert Rev. Anti Infect. Ther..

[7] Motohide S., Takahisa M., Hisateru A., Takashi M., Masaki Y., Yoshiharu I., Hiroshi K., Yuji M., Wataru W., Kazunobu Y. (2006). Novel HIV-1 integrase inhibitors derived from quinolone antibiotics. J. Med. Chem..

[8] Dayam R., Al-Mawsawi L.Q., Zawahir Z., Witvrouw M., Debyser Z., Neamati N. (2008). Quinolone 3-carboxylic acid pharmacophore: Design of second generation HIV-1 integrase inhibitors. J. Med. Chem..

[9] Pasquini S., Mugnaini C., Tintori C., Botta M., Trejos A., Arvela R.K., Larhed M., Witvrouw M., Michiels M., Christ F. (2008). Investigations on the 4-quinolone-3-carboxylic acid motif. 1. synthesis and structure-activity relationship of a class of human immunodeficiency virus type 1 integrase inhibitors. J. Med. Chem..

[10] Sechi M., Rizzi G., Bacchi A., Carcelli M., Rogolino D., Pala N., Sanchez T.W., Taheri L., Dayam R., Neamati N. (2009). Design and synthesis of novel dihydroquinoline-3-carboxylic acids as HIV-1 integrase inhibitors. Bioorg. Med. Chem..

[11] Sato M., Kawakami H., Motomura T., Aramaki H., Matsuda T., Yamashita M., Ito Y., Matsuzaki Y., Yamataka K., Ikeda S., Shinkai H. (2009). Quinolone carboxylic acids as a novel monoketo acid class of human immunodeficiency virus type 1 integrase inhibitors. J. Med. Chem..

[12] Luo Z.G., Zeng C.C., Yang L.F., He H.Q., Wang C.X., Hu L.M. (2009). Synthesis of 6-sulfamoyl-4-oxoquinoline-3-carboxylic acid derivatives as integrase antagonists with anti-HIV activity. Chin. Chem. Lett..

[13] Elmaati T.M.A., El-Taweel F.M. (2004). New trends in the chemistry of 5-aminopyrazoles. J. Heterocycl. Chem..

[14] Sliskovic D.R., Roth B.D., Wilson M.W., Hoefle M.L., Newton R.S. (1990). Inhibitors of cholesterol biosynthesis. 2. 1,3,5-Trisubstituted [2-(tetrahydro-4-hydroxy-2-oxopyran-6-yl)ethyl] pyrazoles. J. Med. Chem..

[15] Zhao Z.G., Wang Z.X. (2007). Halogenation of Pyrazoles using N-Halosuccinimides in CCl4 and in water. Synth. Commun..

[16] Gillespiea R.J., Cliffea I.A., Dawsona C.E., Dourisha C.T., Gaura S., Jordanb A.M., Knighta A.R., Lerpinierea J., Misraa A., Pratta R.M. (2008). Antagonists of the human adenosine A2A receptor. Part 3: Design and synthesis of pyrazolo [3,4-d] pyrimidines, pyrrolo [2,3-d] pyrimidines and 6-arylpurines. Bioorg. Med.Chem. Lett..

[17] Kettler K., Sakowski J., Wiesner J., Ortmann R., Jomaa H., Schlitzer M. (2005). Novel lead structures for antimalarial farnesyltransferase inhibitors. Pharmazie.

[18] Lager E., Andersson P., Nilsson J., Pettersson I., Nielsen E., Nielsen M., Sterner O., Liljefors T. (2006). 4-Quinolone derivatives: High-affinity ligands at the benzodiazepine site of brain GABAA receptors. Synthesis, pharmacology and pharmacophore modeling. J. Med. Chem..

[19] Gould R., Jacob W. (1939). The Synthesis of certain substituted quinolines and 5,6-benzoquinolines. J. Am. Chem. Soc..

[20] He H.Q., Ma X.H., Liu B., Chen W.Z., Wang C.X., Cheng S.H. (2008). A novel high-throughput format assay for HIV-1 integrase strand transfer reaction using magnetic beads. Zhongguo Yao Li Xue Bao.

